# Pharmacokinetic Analysis of the Bioavailability of AQUATURM^®^, a Water-Soluble Curcumin Formulation, in Comparison to a Conventional Curcumin Tablet, in Human Subjects

**DOI:** 10.3390/ph18071073

**Published:** 2025-07-21

**Authors:** Lillian Jabur, Rishi Pandey, Meena Mikhael, Garry Niedermayer, Erika Gyengesi, David Mahns, Gerald Münch

**Affiliations:** 1 Pharmacology Unit, School of Medicine, Western Sydney University, Campbelltown, NSW 2560, Australia; l.jabur@westernsydney.edu.au (L.J.); e.gyengesi@westernsydney.edu.au (E.G.); d.mahns@westernsydney.edu.au (D.M.); 2 Mass Spectrometry Facility, Western Sydney University, Campbelltown, NSW 2560, Australia; r.pandey@westernsydney.edu.au (R.P.); m.mikhael@westernsydney.edu.au (M.M.); 3 School of Science and Health, Western Sydney University, Campbelltown, NSW 2560, Australia; g.niedermayer@westernsydney.edu.au; 4 NICM Health Research Institute, Western Sydney University, Campbelltown, NSW 2560, Australia

**Keywords:** water-soluble curcumin, pharmacokinetics, plasma, curcuminoids, area under the curve, bioavailability

## Abstract

**Background/Objectives:** Curcumin, the principal bioactive component of Curcuma longa, is known for its anti-inflammatory, antioxidant, and neuroprotective properties. Despite its therapeutic potential, curcumin exhibits poor oral bioavailability due to low solubility, rapid metabolism, and limited gastrointestinal absorption. Various delivery systems have been developed to overcome these limitations. This study aimed to evaluate and compare the pharmacokinetic profile of AQUATURM®, a novel, water-soluble curcumin formulation, with that of a widely available commercial curcumin supplement. **Methods:** A randomized, double-blind, two-period crossover study was conducted in 12 healthy adult participants (6 male, 6 female; aged 20–45 years). Each participant received a single oral dose of either AQUATURM® or the comparator product, followed by a 7-day washout period before receiving the alternate treatment. Blood samples were collected at multiple time points over a 12-h period post-dosing. Plasma curcumin concentrations were quantified using ultra-performance liquid chromatography with tandem mass spectrometry (UPLC-MS/MS). **Results:** AQUATURM® achieved a significantly higher systemic exposure compared to the comparator, with a more than 7-fold increase in area under the curve (AUC_0–12_h) and higher peak plasma concentrations (Cmax). AQUATURM® also maintained detectable curcumin levels for the full 12-h observation period, whereas levels from the comparator fell below quantification limits in most participants after 4 h. **Conclusions:** AQUATURM® significantly enhances curcumin bioavailability in humans compared to a standard curcumin formulation. These pharmacokinetic improvements support its potential for greater clinical efficacy and warrant further evaluation in therapeutic setting

## 1. Introduction

Turmeric, derived from the dried rhizome of Curcuma longa, is a member of the Zingiberaceae (ginger) family and has been used for centuries in Asian cuisine and traditional medicine. Its therapeutic effects are primarily attributed to a group of phenolic compounds known as curcuminoids, which include curcumin, demethoxycurcumin, and bisdemethoxycurcumin. Among these, curcumin is the most biologically active and abundant, typically comprising 2–5% of the turmeric root extract.

Curcumin is a natural compound that falls under the diarylheptanoids category, a class of organic compounds characterized by two aromatic rings connected by a seven-carbon chain [1,2]. Curcumin has garnered significant attention in recent years due to its diverse pharmacological properties and potential health benefits [3,4]. These properties position it as a promising candidate for the prevention and treatment of various chronic conditions, particularly those involving systemic or neuroinflammation [5]. Curcumin’s anti-inflammatory action is primarily mediated through modulation of key inflammatory pathways. It has been shown to inhibit enzymes such as cyclooxygenase-2 (COX-2) and 5-lipoxygenase (5-LOX), which drive the synthesis of pro-inflammatory prostaglandins and leukotrienes. Furthermore, curcumin influences gene expression by modulating transcription factors including nuclear factor-kappa B (NF-κB), activator protein-1 (AP-1), signal transducer and activator of transcription 3 (STAT3), and peroxisome proliferator-activated receptor gamma (PPAR-γ). Through these mechanisms, curcumin suppresses the expression of pro-inflammatory cytokines (e.g., TNF-α, IL-1β), chemokines, and adhesion molecules, thereby dampening the inflammatory response [6,7].

Despite its promising therapeutic profile, curcumin’s clinical translation has been hampered by poor water solubility, low gastrointestinal absorption, rapid metabolism, and systemic elimination [8]. To address these challenges, a variety of novel delivery systems have been developed, including nanoparticles, liposomes, micelles, and molecular complexes, all aimed at improving curcumin’s bioavailability and stability, and tested in rodent models for superior bioavailability and CNS permeability [9,10,11,12,13,14], and compared for relative oral bioavailability in humans [15]. Consequently, the assessment of the bioavailability of these formulations in biological matrices, such as blood and tissue, is of paramount importance. Such measurements are pivotal for understanding pharmacokinetics, bioavailability, and distribution of novel curcumin preparations in vivo [15].

One such advancement is AQUATURM^®^, a proprietary curcumin extract developed by LODAAT Pharma. AQUATURM^®^ is a water-dispersible formulation standardized to contain at least 23% total curcuminoids. Its enhanced solubility is achieved through a patented manufacturing process involving particle size reduction (to 45–75 nm) and blending with a unique polysaccharide-based carrier. Unlike many conventional curcumin supplements, AQUATURM^®^ does not require piperine (a black pepper extract) to improve absorption and is reported to be odorless, taste-neutral, and stable in suspension—qualities that enhance its utility in functional foods and dietary supplements.

This pharmacokinetic study was designed to assess whether the improved water solubility of AQUATURM^®^ translates into enhanced systemic bioavailability compared to a widely used commercial curcumin tablet. Using a randomized, double-blind, crossover design in healthy human subjects, we measured plasma curcumin concentrations over a 12 h period following oral ingestion of each formulation. The findings of this study aim to provide critical insights into the pharmacokinetics of AQUATURM^®^ and inform the development of more effective curcumin-based interventions.

## 2. Results

In this study, we compared the pharmacokinetics of a water-soluble curcumin formulation (AQUATURM^®^) with a commercially available comparator, referred to as the Control Curcumin Supplement. A randomized, double-blind, crossover study was conducted in 12 healthy subjects over a 12 h period. Participants received either 450 mg of total curcuminoids from AQUATURM^®^ or from the Control Curcumin Supplement. Both formulations were well tolerated, with no adverse events reported.

**Figure 1:**
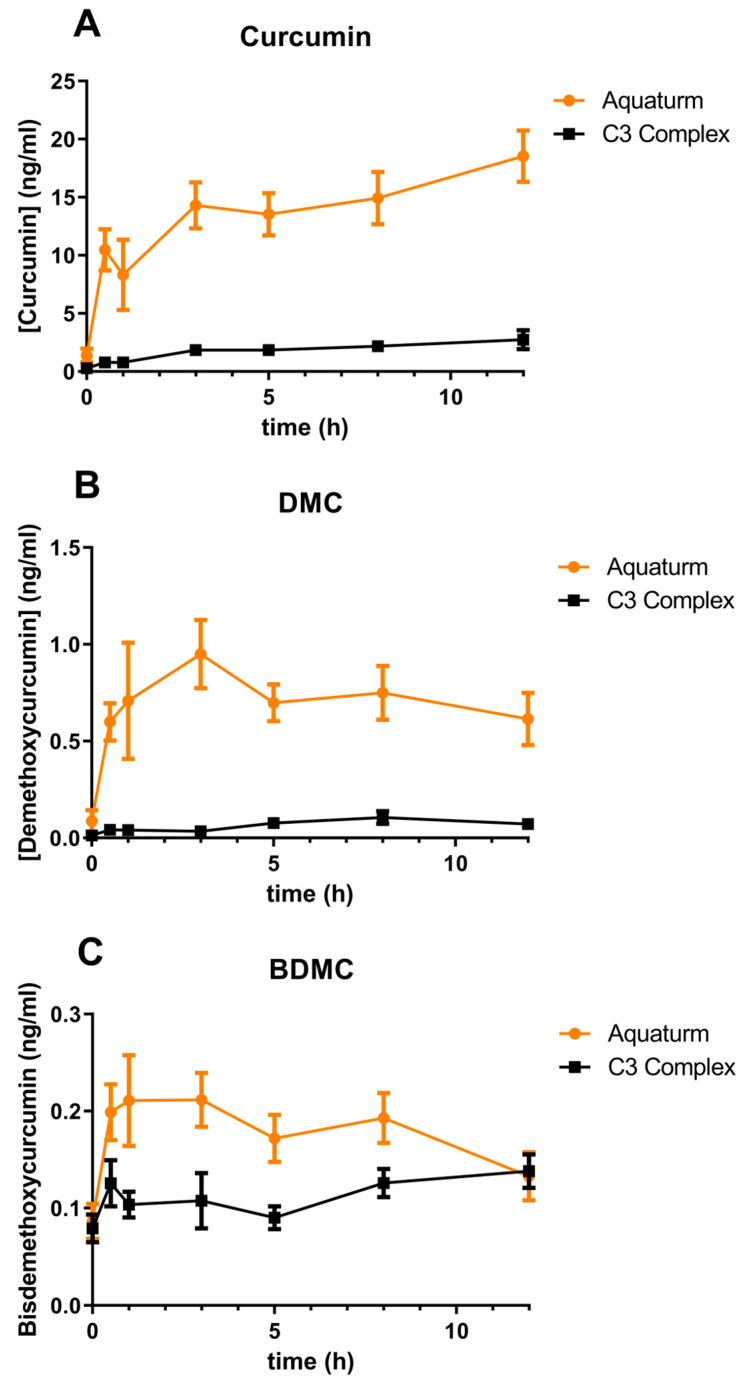
Time course of plasma curcuminoid concentrations over 12 h.

Plasma concentrations of curcumin, demethoxycurcumin, and bisdemethoxycurcumin were measured using UPLC-MS/MS following enzymatic treatment with *Helix pomatia* glucuronidase/sulfatase. This enzymatic hydrolysis step was used to deconjugate sulfate and glucuronide metabolites, thereby enabling quantification of the parent compounds (Figure 1). However, it is important to note that this method may underestimate total curcuminoid levels due to incomplete hydrolysis of complex conjugated metabolites [16].

When the AUC was calculated, AQUATURM^®^ demonstrated a 7.3 times superior bioavailability than that of the Control Curcumin Supplement for curcumin and was in the same range for the other minor curcuminoids (Table 1A). C_max_ was also higher in AQUATURM^®^-treated subjects compared to those given the Control Curcumin Supplementarry Tables S1 and S2 (Table 1B). 

Participants received a single dose of either AQUATURM® or a commercially available Control Curcumin Supplement, each containing 450 mg total curcuminoids. Plasma concentrations of (Figure 1A) curcumin, (Figure 1B) demethoxycurcumin (DMC), and (Figure 1C) bisdemethoxycurcumin (BDMC) were measured at 0, 0.5, 1, 3, 5, 8, and 12 h post-ingestion. Data are presented as mean ± SEM.

## 3. Discussion

An important aspect of pharmacology is the study of drug concentrations in blood and tissues over time to determine whether a drug reaches clinically effective levels at its site of action in the body [17]. The wide array of curcumin formulations available on the market presents a significant challenge for consumers and healthcare professionals when it comes to selecting the most suitable option. Curcumin, a natural compound found in turmeric, has gained substantial attention for its multitude of potential health benefits, including its anti-inflammatory and antioxidant properties [18].

Curcumin’s inherently poor bioavailability—meaning the body has difficulty absorbing and utilizing it effectively—has prompted the development of numerous formulations designed to enhance its delivery and therapeutic potential. These strategies include the use of adjuvants, nanoparticle carriers, and specialized processing techniques. Although some formulations have demonstrated improved absorption and bioactivity, the wide variety and complexity of available products can make it challenging for clinicians to determine the most suitable option for their patients. In this study, we compared a novel curcumin formulation (AQUATURM®) with a widely used reference product, referred to as the “Control Curcumin Supplement”. Our findings show that AQUATURM® exhibits over sevenfold greater bioavailability than the control, achieving plasma concentrations of up to 20 ng/mL and sustaining these levels for up to 12 h. Notably, while other formulations such as Cavamax and Meriva typically peak within 30 min followed by a rapid decline, a single dose of AQUATURM® results in a prolonged elevation of plasma curcumin levels over the 12 h period as also shown in a comparative human study by Purpura et al. [15].

One possible explanation for the sustained high plasma concentration of AQUATURM^®^ is delayed absorption, which may allow elimination to occur concurrently with absorption and distribution. This prolonged presence in the bloodstream could represent a pharmacokinetic advantage over other bioavailable curcumin formulations that are cleared more rapidly.

Previous pharmacokinetic studies have demonstrated similar plasma concentrations of curcumin, DMC, and BDMC when control curcumin was used, validating our method and results. Purpura et al. (in 2018) used an 1800 mg dose of control curcumin, measuring total curcuminoid plasma concentrations of ~2 ng/mL of curcumin, DMC and BDMC [15]. Furthermore, Jäger et al. (in 2014) used an 1800 mg dose of control curcumin, measuring plasma concentrations of ~2 ng/mL of total curcuminoids [19].

However, our study has several limitations that could—at least in part—be addressed through further research. For instance, the single-dose design does not reflect steady-state plasma concentrations that might occur with continuous supplementation. Repeated dosing could lead to accumulation, particularly if the volume of distribution becomes saturated. To address this, future studies should include a multiple-dose regimen, with participants consuming the formulation daily for at least one week prior to pharmacokinetic analysis. Another limitation concerns the use of enzymatic de-glucuronidation and desulfation steps in the analysis. While this approach provides a measure of total curcuminoid bioavailability, it does not differentiate between free (unconjugated) and metabolized forms. This distinction is critical, as emerging evidence suggests that many of curcumin’s biological effects may be exerted primarily by the free form. Consequently, total curcuminoid concentrations may not accurately reflect therapeutic potential, particularly when compared to concentrations used in in vitro studies. An alternative delivery strategy worth exploring is rectal administration, especially for compounds like curcumin that undergo extensive first-pass hepatic metabolism. This route may enhance systemic bioavailability, as the lower rectal veins drain directly into the systemic circulation, bypassing the liver. 

## 4. Materials and Methods

Twelve healthy volunteers (seven males and five females) were recruited for this study. Participants were required to meet the following inclusion criteria: aged between 18 and 65 years, with no history of consuming curcumin-containing supplements or foods (such as curcumin, turmeric, or curry) within 10 days prior to testing; no history of hyperacidity, gastric or duodenal ulcers, or gastrointestinal or gallbladder disorders; no use of blood thinners, anti-thrombotic agents, NSAIDs, blood sugar-lowering agents, H2 blockers, or proton pump inhibitors. Individuals with hyperglycemia, hemophilia, diabetes, or known soy allergies were also excluded. All participants were instructed to avoid consuming pepper for at least two days prior to the study and to refrain from caffeinated beverages on the day of testing. Informed consent was obtained from all participants. The study was approved by the Western Sydney University Human Research Ethics Committee (Approval number: H15160, approval date 24 October 2022).

### 4.1. Study Materials

On two occasions, separated by a 7-day washout period, participants were instructed to orally ingest either one AQUATURM^®^ sachet (providing 450 mg of total curcuminoids) dissolved in 200 mL of water or a standard commercially available curcumin tablet (referred to as the “Control Curcumin Supplement”). AQUATURM^®^ is a proprietary, water-soluble turmeric extract derived from Curcuma longa, standardized to contain 23% total curcuminoids. The control supplement, a Curcumin C3 Complex tablet also delivering 450 mg of total curcuminoids, was sourced from a local health store in Sydney, Australia.

### 4.2. Study Procedure

**Figure 2:**
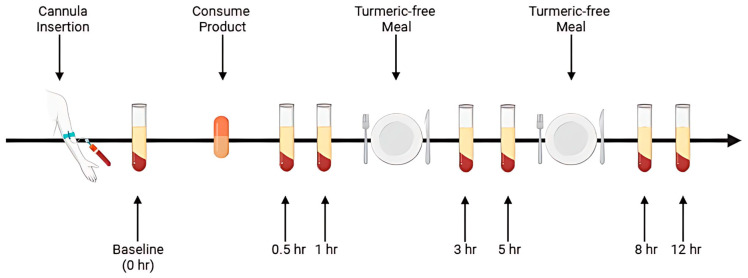
Schematic of the study procedure.

All 12 participants completed both trial sessions, each involving nine blood draws over a single day (Figure 2), under a randomized, double-blind, crossover protocol with a seven-day washout period between formulations. To maintain blinding, the curcumin formulations were coded so that neither the participants nor the investigators knew which product was administered during each session.

On each trial day, subjects arrived at the laboratory in the morning following a 10 h overnight fast (water permitted). A qualified phlebotomist inserted a catheter into a forearm vein to obtain the initial baseline blood sample. Participants then consumed the assigned curcumin formulation, providing 450 mg of total curcuminoids, with water. Subsequent blood samples were collected at 0.5, 1, 3, 5, 8, and 12 h post-supplementation. These time points were selected based on previous pharmacokinetic studies indicating that most digestion and absorption of curcumin occurs within this timeframe [15,19].

After each 4 h and 8 h blood sample collection, a standardized meal free of curcumin was provided. Standardized curcumin-free meals were provided following the 4 h and 8 h blood draws. To ensure protocol adherence, all participants remained in the laboratory for the full duration of each study session.

The study began with administration of the AQUATURM^®^ formulation, followed by a 7-day washout period. The Control Curcumin Supplement^®^ tablet was then administered using the same 12 h procedure. Upon arrival at the clinic, a cannula was inserted, and a baseline blood sample was collected prior to curcumin ingestion. Following ingestion, blood samples were collected at six time points over a 12 h period. Participants received a turmeric-free meal after the 1 h and 5 h blood draws.

### 4.3. Sample Collection

A qualified phlebotomist inserted a cannula into a suitable vein in the antecubital fossa of each participant. Blood samples were collected into 6 mL EDTA tubes at baseline (pre-ingestion) and at 0.5, 1, 3, 5, 8, and 12 h following curcumin ingestion. The samples were centrifuged at 4 °C for 10 min at 2300 RPM. The plasma (upper layer) was then separated and stored at −80 °C until further analysis.

### 4.4. Sample Preparation

The plasma samples were prepared according to a modified procedure based on Cuomo et al. [20]. Plasma samples were thawed at room temperature prior to analysis. Stock solutions of all analytes and deuterated internal standards were prepared by dissolving 10 mg of each compound in 1 mL of methanol. For extraction, a 200 µL aliquot of plasma was transferred into an Eppendorf tube, followed by the addition of 1 µL acetic acid, 2 µL of a 100 ng/mL working solution of deuterated curcumin (CD6) as the internal standard (final concentration: 1 ng/mL), and 100 µL of a solution containing 1000 U of β-glucuronidase/sulfatase (EC 3.2.1.31) from *Helix pomatia* (Sigma, St. Louis, MO, USA) prepared in 0.1 M phosphate buffer (pH ~ 4.2). The mixture was vortexed thoroughly and incubated at 37 °C for 1 h to enzymatically hydrolyze phase II curcuminoid conjugates.

Following incubation, samples were heated to 70 °C for 10 min, after which 700 µL of ethyl acetate was added as the extraction solvent. The mixture was vortexed for 5 min, sonicated for 15 min, and centrifuged at 14,000 RPM for 6 min at 4 °C. The upper organic layer was carefully transferred to a new Eppendorf tube. This extraction process was repeated three times, yielding a pooled supernatant volume of approximately 1500 µL.

The combined extract was evaporated to dryness at 30 °C under reduced pressure using a centrifugal concentrator. Dried residues were reconstituted in 200 µL of methanol and transferred to total recovery glass vials, and 5 µL aliquots were injected into the UPLC-MS/MS system for analysis [20].

### 4.5. Chromatographic Analysis of the Curcuminoids

Blood plasma samples and standards were analyzed by UPLC-MS/MS. The UPLC-MS/MS system utilized was a Waters ACQUITY UPLC I-Class system coupled to a SCIEX triple quadrupole 7500 QTRAP system equipped with an OptiFlow^®^ Pro Ion Source. A Waters ACQUITY™ UPLC I-Class autosampler was employed to inject 5 μL of sample using the partial loop method. Analyte separation was achieved with a Waters ACQUITY^TM^ UPLC HSS T3 (1.8 μm, 2.1 × 100 mm) at a temperature of 35 °C and flow rate of 0.200 mL/min. Mobile phases A (Milli-Q water with 0.1% formic acid) and B (acetonitrile with 0.1% formic acid) were run with a linear gradient of 50% B to 65% at 3.0 min and a linear gradient to 100% at 5.0 min, and B was held at 100% until 8.0 min, at which point the gradient changed to initial conditions (50% B) for equilibration until 10 min. Ionization was achieved by utilizing the OptiFlow^®^ Pro Ion Source in positive scanning mode at 450 °C with an ion spray voltage of 2000 V. The following optimized ion source parameters were utilized: curtain gas 40 psi, ion source gas 1: 50 psi, ion source gas 2: 70 psi, CAD gas 9. Scheduled multiple reaction monitoring (MRM) was performed for analytes using optimized entrance potential (EP), collision energies (CEs), and collision cell exit potentials (CXPs) (Supplementary Tables S1 and S2). The analyte precursor ions, [M + H^+^], and three of their product ion fragments were monitored to quantify and confirm the identity of the analytes (Supplementary Tables S1 and S2). Compound MRM transitions and optimal CE and CXP voltages for each transition were determined through direct injection. Characteristic Q3 product ions were selected (curcumin: *m*/*z* = 177, demethoxycurcumin: *m*/*z*: 147, bisdemethoxycurcumin: *m*/*z*: 119, and deuterated d6-curcumin: *m*/*z*: 180, Figure S1, Table S1).

The full method validation will be published in a separate manuscript and details will only be mentioned here briefly.

Limits of detection (LODs) and limits of quantification (LOQs): The limits of detection (LODs) and limits of quantification (LOQs) were determined using neat standards, with LOD defined as a signal-to-noise ratio greater than 3, and LOQ as a signal-to-noise ratio greater than 10. The LOD values for curcumin (Curc), bisdemethoxycurcumin (BDMC), and demethoxycurcumin (DMC) were found to be 0.00045 ng/mL, 0.00038 ng/mL, and 0.000705 ng/mL, respectively. The corresponding LOQ values were determined to be 0.0015 ng/mL, 0.0012 ng/mL, and 0.00235 ng/mL, respectively. Precision: The intra-day and inter-day coefficients of variation (CV%) across low-level spiked recovery samples (*n* = 4–5 per day) were below 15% for all analytes (curcumin, DMC, BDMC). (Table S2) Accuracy: Calculated concentrations of recovery samples were within ±20% of the nominal concentrations, indicating good accuracy. Linearity: The method demonstrated linear response across the relevant concentration range (0.1–50 ng/mL) with R^2^ > 0.99 for all analytes, as assessed from internal calibration curves. Retention time stability: Retention time drift was minimal (±0.1 min), and internal standard-normalized responses were consistent across runs (Table S1). A sample chromatogram is displayed in Supplementary Figure S1.

### 4.6. Pharmacokinetic Analysis of Curcuminoid Plasma Concentrations

Pharmacokinetic parameters following oral administration of the curcumin formulations were analyzed using GraphPad Prism 5. The maximum observed plasma concentration (Cmax) was determined directly from the mean plasma concentration–time profiles. The area under the curve (AUC 0–12 h) was calculated using the trapezoidal (additive) method over the 12 h sampling period. The elimination half-life (t½) could not be determined, as plasma curcuminoid concentrations did not show a declining trend within the 12 h timeframe.

## 5. Conclusions

Curcuminoids are natural compounds found in turmeric, known for their anti-inflammatory and antioxidant properties. However, their therapeutic potential has been limited by inherently poor bioavailability. In this study, we demonstrated that AQUATURM^®^—a novel curcumin formulation—is approximately seven times more bioavailable than a widely used commercial curcumin supplement. Moreover, the individual curcuminoid components of AQUATURM^®^, including curcumin and demethoxycurcumin, also showed significantly improved bioavailability. These findings represent a promising step toward realizing the full therapeutic potential of curcuminoids. Further research is needed to evaluate the clinical relevance of this enhanced bioavailability. Key areas for future investigation include long-term safety, efficacy across diverse populations and disease contexts—particularly (neuro)inflammatory conditions—and direct comparisons with other advanced curcumin formulations. If confirmed, AQUATURM^®^ may offer a therapeutic option capable of achieving and maintaining clinically effective plasma concentrations over extended periods.

## Figures and Tables

**Table 1:** (**A**) Comparison of the area under the curve between 0 and 12 h (AUC _12h_). (**B**) Comparison of the maximal concentration (C_max_).

(**A**)				
**Curcuminoid**	**AQUATURM^® ^** **(AUC_12h_)**	**Control Curcumin Tablet (AUC_12h_)**	**Ratio of AUCs** **(Drug/Control)**	**Significance**
Curcumin	167.7	22.85	7.33	<0.001
DMC	8.70	0.85	10.2	<0.001
BDMC	2.17	1.36	1.59	<0.001
Total Curcuminoids	178.5	25.1	7.12	<0.001
(**B**)				
**Curcuminoid**	**AQUATURM^® ^** **(C_max_ in ng/mL)**	**Control Curcumin Tablet (C_max_ in ng/mL)**	**Ratio of C_max_** **(Aquaturm/Control Supplement)**	
Curcumin	18.53	2.74	6.76	
DMC	0.83	0.07	11.85	
BDMC	0.21	0.15	1.40	

Note that significances and total curcuminoid concentrations are not displayed in Table 1B, as the C_max_ time points correspond to different amounts of the formulations.

## Data Availability

Data are contained within the article and Supplementary Materials.

## Supplementary Materials

The following supporting information can be downloaded at https://www.mdpi.com/article/10.3390/ph18071073/s1: Figure S1: Chromatogram showing the retention times of the 3 curcuminoids plus deuterated curcumin. Figure S2: Standard curves. Table S1: Mass spectrometry parameters for curcuminoids. Table S2: Gradient parameters for the UPLC method.
