# Supplementary material for: Pharmacokinetic Analysis of the Bioavailability of AQUATURM®, a Water-Soluble Curcumin Formulation, in Comparison to a Conventional Curcumin Tablet, in Human Subjects
Source: Pharmaceuticals (Basel). 2025 Jul 21;18(7):1073. doi: 10.3390/ph18071073 (PMC12298949; doi:10.3390/ph18071073)
Supplement: Supplementary file 1 [file pharmaceuticals-18-01073-s001.zip › SM Figures.pdf]

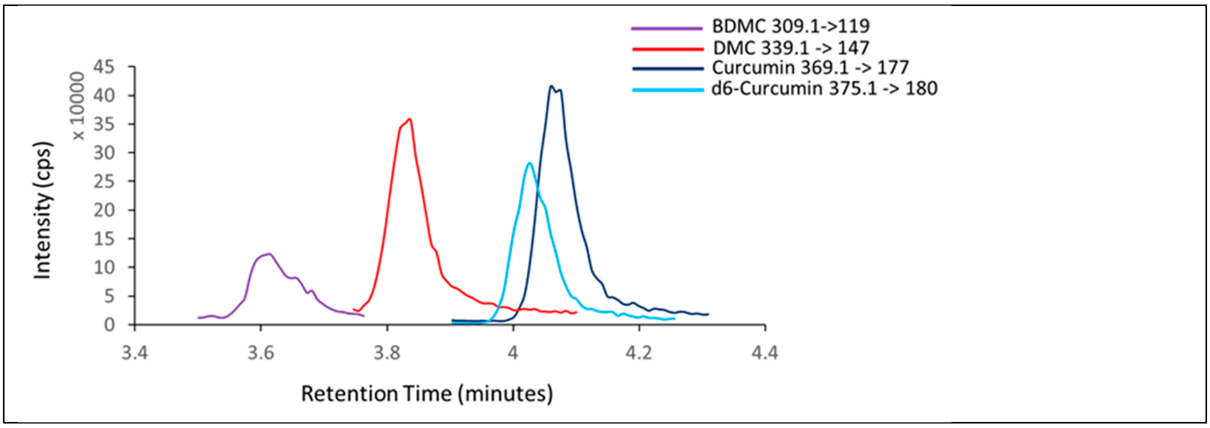

Figure S1. Chromatogram showing the retention times of the 3 curcuminoids plus deuterated curcumin including the MW of the compound and the selected fragment ion for quantification

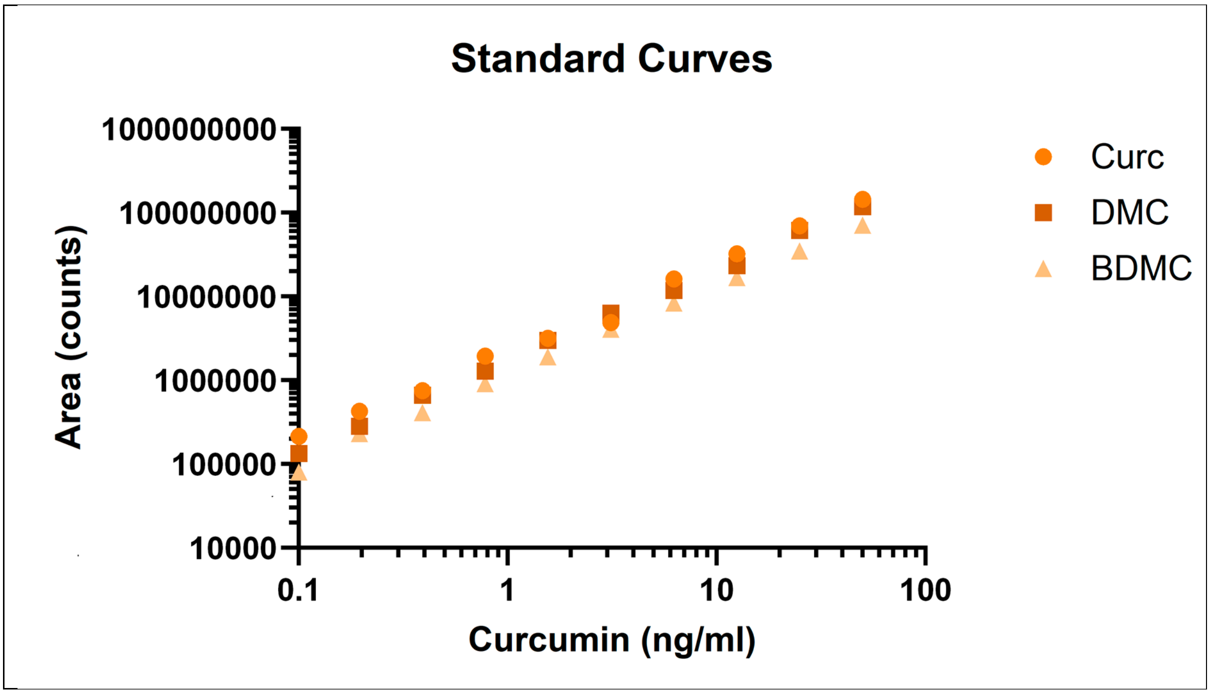

Figure S2. Linear standard curves for the quantification of three curcuminoids (curcumin, demethoxycurcumin, and bisdemethoxycurcumin) in the concentration range of 0.1–50 ng/mL. The calibration curves were constructed using peak area of the selected Q 3 ion versus concentration, showing excellent linearity with correlation coefficients (R^2) of ⩾0.99 for all three compounds.
