# Supplementary material for: Pharmacokinetic Analysis of the Bioavailability of AQUATURM®, a Water-Soluble Curcumin Formulation, in Comparison to a Conventional Curcumin Tablet, in Human Subjects
Source: Pharmaceuticals (Basel). 2025 Jul 21;18(7):1073. doi: 10.3390/ph18071073 (PMC12298949; doi:10.3390/ph18071073)
Supplement: Supplementary file 1 [file pharmaceuticals-18-01073-s001.zip › SM Tables.pdf]

Table S1: Mass spectrometry parameters for curcuminoids

Mass spectrometry parameters used for the detection and quantification of curcuminoids. Thetable includes analyte name, retention time, precursor ion, Q1 and Q3 masses, and optimized voltages for entrance potential (EP), collision energy (CE), and collision cell exit potential (CXP).

**Analyte**	**Retention time (min)**	**Precursor ion**	**Q1 mass (Da)**	**Q3 mass (Da)**	**EP (V)**	**CE (V)**	**CXP (V)**
**Curcumin**	3.9	$[M+H]^+$	369.1	177*	10	33	9
**Curcumin**	3.9	$[M+H]^+$	369.1	285	10	26	21
**Curcumin**	3.9	$[M+H]^+$	369.1	145	10	47	8
**DMC**	3.7	$[M+H]^+$	339.1	147*	10	34	10
**DMC**	3.7	$[M+H]^+$	339.1	177	10	28	6
**DMC**	3.7	$[M+H]^+$	339.1	119	10	22	9
**BDMC**	3.5	[M+H]+	309.1	119*	10	48	8
**BDMC**	3.5	[M+H]+	309.1	147	10	30	10
**BDMC**	3.5	[M+H]+	309.1	225	10	21	8
**d6-Curcumin**	3.9	$[M+H]^+$	375.1	180*	10	33	8
**d6-Curcumin**	3.9	$[M+H]^+$	375.1	291	10	26	8

Table S2: Gradient parameters for the UPLC method

Gradient elution program used in the UPLC method for the separation of curcuminoids. Solvent A was water with 0.1% formic acid, and Solvent B was acetonitrile with 0.1% formic acid. The gradient was applied over a 10-minute run with specified changes in solvent composition and curve settings for pump control.

**Time (min)**	**Solvent A**	**Solvent B**	**Curve**
Initial	50	50	6
3	35	65	6
5	0	100	6
8	50	50	11
10	50	50	11
